# Legumes Modulate Allocation to Rhizobial Nitrogen Fixation in Response to Factorial Light and Nitrogen Manipulation

**DOI:** 10.3389/fpls.2019.01316

**Published:** 2019-11-05

**Authors:** Colleen A. Friel, Maren L. Friesen

**Affiliations:** ^1^Department of Plant Biology, Michigan State University, East Lansing, MI, United States; ^2^BEACON Center for Evolution in Action, Michigan State University, East Lansing, MI, United States

**Keywords:** mutualism, symbiosis, resource exchange, optimal partitioning theory, nitrogen fixation

## Abstract

The costs and benefits that define gain from trade in resource mutualisms depend on resource availability. Optimal partitioning theory predicts that allocation to direct uptake *versus* trade will be determined by both the relative benefit of the resource acquired through trade and the relative cost of the resource being traded away. While the costs and benefits of carbon:nitrogen exchange in the legume–rhizobia symbiosis have been examined in depth with regards to mineral nitrogen availability, the effects of varying carbon costs are rarely considered. Using a growth chamber experiment, we measured plant growth and symbiosis investment in the model legume *Medicago truncatula* and its symbiont *Ensifer medicae* across varying nitrogen and light environments. We demonstrate that plants modulate their allocation to roots and nodules as their return on investment varies according to external nitrogen and carbon availabilities. We find empirical evidence that plant allocation to nodules responds to carbon availability, but that this depends upon the nitrogen environment. In particular, at low nitrogen—where rhizobia provided the majority of nitrogen for plant growth—relative nodule allocation increased when carbon limitation was alleviated with high light levels. Legumes’ context-dependent modulation of resource allocation to rhizobia thus prevents this interaction from becoming parasitic even in low-light, high-nitrogen environments where carbon is costly and nitrogen is readily available.

## Introduction

Interactions between plants and microbial symbionts are major drivers of global nutrient cycles and play vital roles in the productivity of natural and agricultural ecosystems (van der Heijden et al., 2008). Microbial symbionts can supply plants with both nitrogen and phosphorus, essential nutrients that commonly limit plant growth (Erisman et al., 2013). An ancient example of such a symbiosis is the interaction between plants and mycorrhizal fungi, where fungi colonize plant roots and use their hyphal network to supply water, phosphorus, and other mineral nutrients to plants in exchange for 5 billion tons of photosynthetically fixed carbon each year (Bago et al., 2000). Similarly, soil bacteria known as rhizobia can colonize plant roots and induce the formation of nodules, inside of which the rhizobia fix an estimated 40 million tons of plant-inaccessible nitrogen from the atmosphere in exchange for photosynthetic carbon and other nutrients required for growth and metabolism (Udvardi and Poole, 2013). While these interactions are generally regarded as mutualistic, with the symbiosis increasing fitness for both partners (Bronstein, 2015), theory predicts that symbioses exist along a gradient from mutualism to parasitism depending on the environmental context (Bronstein, 1994; Johnson et al., 1997; Bronstein, 2001; Neuhauser and Fargione, 2004).

One key factor that may shift an interaction along the mutualism–parasitism continuum is the availability of the traded resources in the environment. Symbionts are predicted to shift from mutualism toward parasitism when the resource they supply is abundant in the soil, and, thus, the benefit to the host of obtaining it from the symbiont is reduced (Bronstein, 1994; Neuhauser and Fargione, 2004). With high levels of nitrogen fertilizer use and nitrogen deposition across ecosystems (Foley et al., 2005), there is concern that nitrogen-fixing symbioses between plants and microbes such as rhizobia may break down. Even a single growing season of fertilizer application is sufficient to change the composition of the rhizobia population in soil (Simonsen et al., 2015). Long-term nitrogen addition experiments have shown that prolonged nitrogen fertilization leads to the evolution of less effective rhizobia in the *Trifolium*-*Rhizobium* symbiosis (Weese et al., 2015). This partner quality decline may be linked to evolutionary differentiation in the symbiotic plasmid (Klinger et al., 2016). However, in the presence of externally supplied nitrogen, the plant may be able to minimize the costs associated with less beneficial rhizobia by reducing or eliminating its allocation of resources to the microbes.

The way a plant allocates resources to microbial symbionts such as rhizobia can be considered in the framework of biological market theory (Schwartz and Hoeksema, 1998). The plant can allocate its resources in two distinct ways: it can increase root biomass to take up nitrogen directly from the soil, or it can increase photosynthesis to acquire carbon to trade for nitrogen with rhizobia. The cost-benefit analysis of trade *versus* direct uptake depends on the availability of both traded resources. Optimal partitioning theory predicts that the plant will allocate biomass to the part of the plant that acquires the resource that is most limiting to the plant (Thornley, 1972; Bloom et al., 1985). In this case, each partner will specialize in acquiring the resource for which it has a comparative advantage and trade to acquire the other resources, and both partners will acquire more total resources than they would in isolation (Schwartz and Hoeksema, 1998). When trade is beneficial, an organism increases its own potential fitness by engaging in trade, and mutualisms can readily evolve, essentially as by-product mutualisms. However, if the resource being traded away is not available in excess, or if the resource being traded for is abundant in the environment and/or cheap to obtain, the fitness gain from trade may become negative (Johnson et al., 1997; Schwartz and Hoeksema, 1998). In this context, optimal partitioning theory predicts that, under high mineral nitrogen levels, the plant will downregulate allocation to symbiosis—in extreme cases terminating the relationship entirely if they are able to. However, a plant’s optimal allocation to nitrogen uptake will also depend critically upon both the carbon cost of each uptake strategy as well as the carbon available to the plant.

There is a large body of empirical evidence for shifts in allocation and context-dependent benefits in response to nutrient availability for the nutrient supplied by the microbe in plant-microbe nutritional symbioses, but very few studies have investigated the effects of factorially manipulating both traded resources. In particular, the negative effects of soil nitrate, the most commonly available form of soil nitrogen, on nodulation have long been reported in the literature (Streeter and Wong, 1988; Lucinski et al., 2002; Glyan’ko et al., 2009), though the magnitude of the effect of nitrate on nodulation may be strongly affected by genotype-by-genotype interactions (Heath et al., 2010). In contrast, relatively little is known about the effects of light on these interactions. The studies that do exist have found wildly varying results: Houx et al. (2009) found that three species of *Desmodium* all exhibit reduced plant biomass and total nodule number with shade, but that the ratio of nodule biomass to root biomass and plant nitrogen content does not change. *Trifolium repens* reduced total nodule biomass in shaded conditions, but this was mostly explained by reduced root biomass (Chu and Robertson, 1974). Various studies in soybean have shown conflicting results: shading increases nodule biomass and decreases efficiency (Santos et al., 1997), decreases nodule biomass and increases efficiency (Araujo et al., 2018), and decreases nodule biomass but does not affect efficiency (Hansen et al., 1990).

The literature regarding interactions between nitrogen and carbon availability is even sparser. Lau et al. (2012) manipulated N-P-K fertilizer and light levels and found that *Bradyrhizobium japonicum* nodulation on *Glycine max* (soybean) was significantly decreased by low light levels, but this study did not detect an effect of fertilizer on nodulation. *Bradyrhizobium* increased plant biomass in the low nutrient, high light conditions but had no effect on aboveground plant biomass in low nutrient, low light conditions or in any high nutrient conditions (Lau et al., 2012), making it difficult to interpret this study in the framework of market theory. Similarly, Regus et al. (2015) manipulated KNO_3_ application and light regime (by season of growth in the greenhouse) in the *Lotus strigosus*–*Bradyrhizobium* symbiosis. They found that nitrogen fertilization eliminated plant growth benefits from rhizobia in the fall when there were lower light levels, while nitrogen fertilization reduced but did not eliminate plant growth benefits from rhizobia in the winter when there were higher light levels. Nitrogen decreased nodule biomass in both seasons, while it decreased nodule number in fall but not winter, consistent with the idea that low light makes carbon more expensive and thus reduces the overall investment in symbiosis (Regus et al., 2015). These results suggest that there may be interactions between the availability of carbon and nitrogen, though it is impossible to rule out other environmental factors that varied between seasons. Thus, these initial results highlight the need for a highly controlled analysis of the interactions between carbon and nitrogen availabilities and their effects on plant investment in trade with nitrogen-fixing rhizobia.

In this study, we examined changes in plant biomass allocation and trade with rhizobia in response to variation in both light level (a proxy for carbon availability; Farquhar et al., 1980) and soil mineral nitrogen. We used the model legume *Medicago truncatula* and its rhizobial partner *Ensifer medicae* WSM419 grown under controlled conditions with factorial light and nitrogen resource manipulation. We hypothesized that plants would allocate resources optimally according to optimal partitioning theory, leading to three predictions: (1) plants will allocate more resources to acquiring the limiting nutrient (nitrogen or carbon) as a function of external inputs, (2) plants will allocate relatively more resources to nodules than to roots when soil nitrogen is low and thus the return on investment for root allocation is reduced relative to high soil nitrogen, and (3) when soil nitrogen is low, plants at high light will invest highly in nodules but, at low light carbon, scarcity will reduce nodule allocation. Our central hypothesis is that the availability of carbon will modulate the relative return on investment from roots *versus* nodules and predict that plants will invest more in trade with rhizobia for fixed nitrogen when carbon is readily available.

## Materials and Methods

### Pot Preparation

SC10 Cone-tainers (Stuewe and Sons Inc., Corvallis, OR, USA) were filled with medium vermiculite. The pots had a ⅜” cotton wick leading to an opaque 50 ml reservoir to ensure constant access to liquid and nutrients. Prior to planting, we added 25 ml of deionized water to each pot, autoclaved once for 60 min then fertilized with 25 ml of Fahraeus solution with 8 or 80 mg/L N (as NH_4_NO_3_). We then autoclaved the pots twice more for 60 min with approximately 24 h between each run.

### Seedling Preparation

We scarified seeds of the model legume *M. truncatula* genotype A17 (Young et al., 2011) with 600 grit sandpaper and sterilized them in full strength commercial bleach (8.25% NaHClPO_3_) for 3 min, followed by six rinses with sterile deionized water. We incubated the seeds in sterile deionized water for 3 h at room temperature then re-sterilized imbibed seeds in 10% bleach for 30 s. After six rinses with sterile deionized water, seeds were incubated in sterile deionized water for 48 h at 4°C. We changed the water once every 12 h. After 48 h, we transferred seeds to sterile petri dishes sealed with Parafilm and germinated them at room temperature in the dark for 48 h. Seedlings with radicles at least 1 cm long were transplanted into prepared pots. After planting, the plants were fully randomized and grown at 25°C with a 16 h day/8 h night cycle at approximately 150 µmol•m^−2^•s^−1^ to encourage uniform seedling establishment. Seedlings were misted daily with sterile deionized water to keep the radicles moist. After 5 days, plants were transferred to a high light growth chamber where half of the plants received full irradiation (400 µmol•m^−2^•s^−1^), and half were shaded with Sun Mesh Sunblock shade cloth to 200 µmol•m^−2^•s^−1^. Full irradiance plants were grown in one rack while shaded plants were grown in a separate rack, with racks placed side by side in the center of the growth chamber to minimize edge effects. All plants were grown with equal spacing that prevented shading by other plants. All plants were grown at 25°C with a 16 h day/8 h night cycle. We grew 8–11 replicate plants per experimental treatment combination.

### Rhizobia Preparation

Cultures of *E. medicae* WSM419 (Reeve et al., 2010) were grown for 48 h in TY broth with 3.4 mM CaCl_2_ at 30°C shaking at 200 RPM. Cell density was determined by measuring the OD600 of the culture using a NanoDrop. After 3 days of acclimation to the new growth chamber conditions, rhizobia inoculated plants received 1 ml of 10^7^ CFU/ml suspended in ½x phosphate buffered saline (PBS; http://cshprotocols.cshlp.org/content/2006/1/pdb.rec8247), and rhizobia-free plants received 1 ml of sterile ½x PBS. Sterilities of the mock inoculum and cell count of the rhizobial inoculum were checked using spot plating and serial dilution on TY agar.

### Plant Growth and Harvest

Plants were watered from below with 25 ml of sterile Fahraeus nutrient solution with 8 or 80 mg/L nitrogen (as NH_4_NO_3_) whenever the reservoirs ran dry (at least twice a week). After 4 weeks of growth, plants were harvested. Nodules were plucked and counted, and tissue was dried at 60°C for 1 week. We measured root, shoot, and nodule dry weights and nodule number. No uninoculated plants developed nodules, indicating that there was no rhizobial contamination of uninoculated plants.

### Statistical Analysis

To test the effects of soil nitrogen and light availability on nodule biomass and the ratio of nodule biomass to root biomass, we used a linear model ANOVA with type II sum of squares (car package, R 3.5.1) with nitrogen and light main effects and nitrogen by light interactions as fixed effects. Nodule biomass and the nodule:root ratio were ln-transformed to improve normality. We tested the effects of nitrogen and light on nodule number using a generalized linear model with a Poisson distribution and ANOVA with type II sum of squares (car package, R 3.5.1).

To test the relationship between nodule biomass and shoot biomass across nitrogen and light conditions, we used a linear model ANOVA with type II sum of squares (car package, R 3.5.1) with nitrogen, light, nodule biomass, and all their interactions as fixed effects. Shoot biomass and nodule biomass were ln-transformed to improve normality.

To test the effects of nitrogen, light, and rhizobial inoculations on shoot biomass, root biomass, and the root:shoot ratio, we used a linear model ANOVA with type II sum of squares (base R and car package, R 3.5.1) with light, soil nitrogen, and rhizobial status and all interactions as fixed effects. Shoot biomass and root biomass were ln-transformed to improve normality. In all cases, to determine whether group means were significantly different, we conducted *post hoc* testing with the Tukey test at a significance level of 0.05 (lsmeans package, R 3.5.1). We note that one plant was an extreme outlier and was removed from the analysis but is still in the raw data (**Supplementary Data Sheets 1** and **2**). Removal of this plant weakened the statistical patterns we observed.

## Results

### Both Light and Nitrogen Affect Total Investment in Nodules

**Figure 1:**
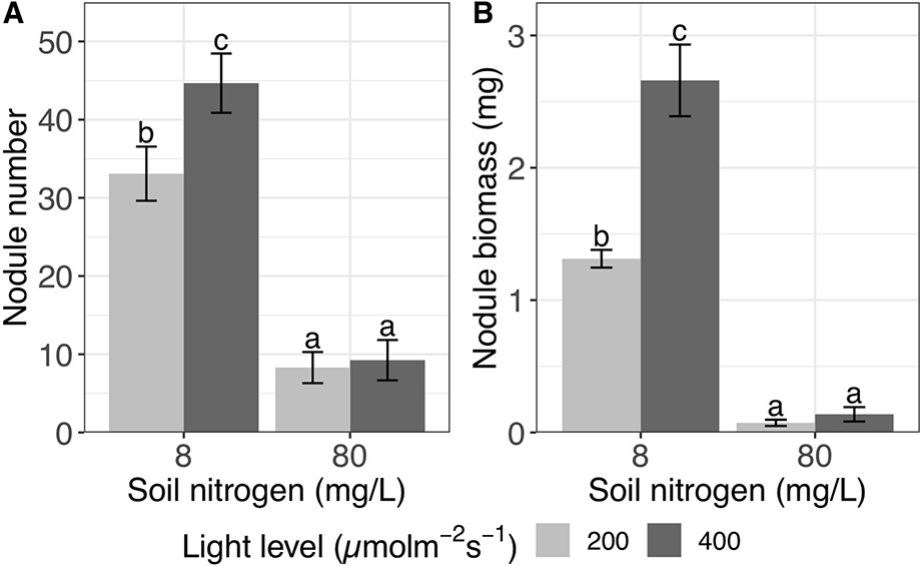
Effects of light and nitrogen on total nodulation. **(A)** Nodule number and **(B)** nodule biomass in inoculated *Medicago truncatula* plants in response to changing soil nitrogen and light availability levels. Error bars represent +/− one standard error (8–11 replicates). Note that nodule biomass was ln-transformed to improve normality in the ANOVA but is represented here without transformation for ease of interpretation. Bars with the same letter within an individual panel do not significantly differ after Tukey *post hoc* testing (*P* > 0.05).

High light increased nodule number by 32% overall (*P* < 0.0001), while high soil nitrogen decreased nodule number by 77% overall (*P* < 0.0001; **Table 1**). We did not detect a significant interactive effect of light and nitrogen on nodule number (*P* > 0.05; **Table 1**). This was surprising since high light significantly increases nodule number by 35% at low soil nitrogen (*P* < 0.001) but non-significantly by 11% at high soil nitrogen (*P* > 0.05; **Figure 1A**). It seems likely that this statistical anomaly is caused by the relatively small effect size of light overall.

**Table 1:** ANOVAs summarizing the effects of light, nitrogen, and their interaction (*) on nodulation. Bold indicates statistically significant effects (*P* < 0.05).

	Nodule number		Nodule biomass	
	χ^2^	*P*	*F_(1,34)_*	*P*
Light (L)	**16.5**	**4.9e−05**	**25.1**	**1.7e−05**
Nitrogen (N)	**375**	**<2e−16**	**342**	**<2.2e−16**
L*N	1.19	0.28	**14.1**	**6.5e−04**

We detected a significant interaction between the effects of light and nitrogen on nodule biomass (*P* < 0.0001; **Table 1**). High soil nitrogen significantly reduced nodule biomass by 95% regardless of light level (*P* < 0.001 at both light levels; **Figure 1B**). However, at low soil nitrogen, high light increased nodule biomass by 103% (*P* < 0.001), while high light did not significantly increase nodule biomass at high soil nitrogen (*P* > 0.05; **Figure 1B**).

### Both Light and Nitrogen Affect Symbiosis Formation and Investment in Rhizobia *Versus* Direct Uptake, but Not Return on Investment

**Figure 2:**
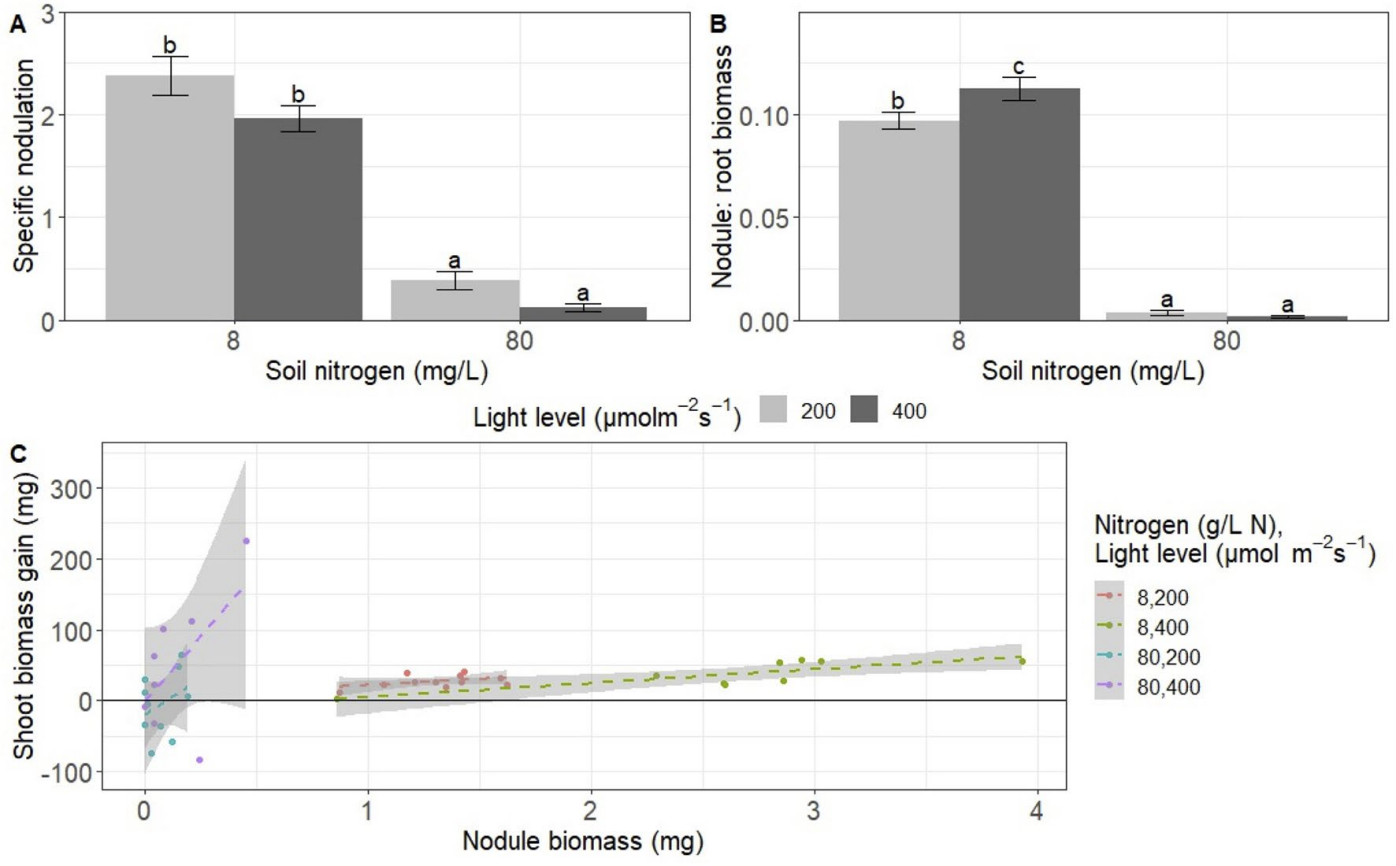
Effects of light and nitrogen on investment in trade *versus* direct uptake and return on investment in trade. **(A)** Specific nodulation (nodule number per milligram root biomass), **(B)** nodule:root biomass (mg nodule biomass per mg root biomass), and **(C)** symbiosis efficiency mg shoot gained per mg invested in rhizobia). Each point represents an individual plant (8–11 replicates). Bars with the same letter within a panel do not significantly differ after Tukey post hoc testing (P > 0.05).

We also assessed measures of nodulation scaled by root biomass to directly test how resource availability affected relative allocation of biomass between trade and direct uptake. High light decreased specific nodulation (nodule number per mg of root biomass) by 31% overall (*P* < 0.01; **Table 2**), though there were no significant differences in specific nodulation when all pairwise combinations were tested during Tukey testing (*P* > 0.05 at low nitrogen and *P* > 0.05 at high nitrogen; **Figure 2A**). High nitrogen decreased specific nodulation by 88% overall (*P* < 0.0001; **Table 2**, **Figure 2A**).

**Table 2:** ANOVAs summarizing the effects of light, nitrogen, and their interaction (*) on nodulation scaled by root biomass. Bold indicates statistically significant effects (*P* < 0.05).

	Specific nodulation		Nodule:root biomass	
	*F_(1,34)_*	*P*	*F_(1,34)_*	*P*
Light (L)	9.05	**0.005**	3.80	0.060
Nitrogen (N)	318	**<2e−16**	**858**	**<2.2e−16**
N*L	0.560	0.46	**5.77**	**0.02**

We detected a significant interaction between the effects of light and nitrogen on nodule:root biomass (mg nodule biomass per mg root biomass) (*P* < 0.05; **Table 1**). High nitrogen significantly decreased nodule:root biomass by approximately 97% regardless of light level (*P* < 0.001 for both light levels; **Figure 2B**). High light increased nodule:root biomass by 16% at low nitrogen (*P* < 0.05) but did not have a significant effect on nodule:root biomass at high nitrogen (*P* > 0.05; **Figure 2B**).

We also examined the relationship between nodule biomass and shoot biomass gain from rhizobia to assess the return on investment from rhizobia. We detected a significant interaction between the effects of nitrogen and nodule biomass on shoot biomass gain from rhizobia, or shoot biomass minus mean biomass of control plants per condition (*P* < 0.01; **Table 3**). We did not detect a significant main effect of light, nitrogen, an interaction between the effects of light and nitrogen, or a three-way interaction between light, nitrogen, and nodule biomass (**Table 3**). The significant nitrogen by nodule biomass interaction term indicated that the slope of the linear regression of shoot biomass gain on nodule biomass differed based on nitrogen treatment (**Figure 2C**). However, neither slope was significantly different from zero (*P* > 0.05 for low nitrogen and *P* > 0.05 for high nitrogen).

**Table 3:** ANCOVA summarizing the effects of light, nitrogen, and their interaction (*) on the relationship between nodule biomass and shoot biomass gain. Bold indicates statistically significant effects (*P* < 0.05).

	Shoot biomass gain	
	*F_(1,30)_*	*P*
Light (L)	0.910	0.35
Nitrogen (N)	2.05	0.16
Nodule biomass (NB)	2.67	0.11
L*N	0.540	0.47
L*NB	0.056	0.81
N*NB	**9.62**	**0.004**
L*N*NB	0.446	0.51

### Nitrogen, Light, and Rhizobia Affect Relative Allocation Between Root and Shoot

**Figure 3:**
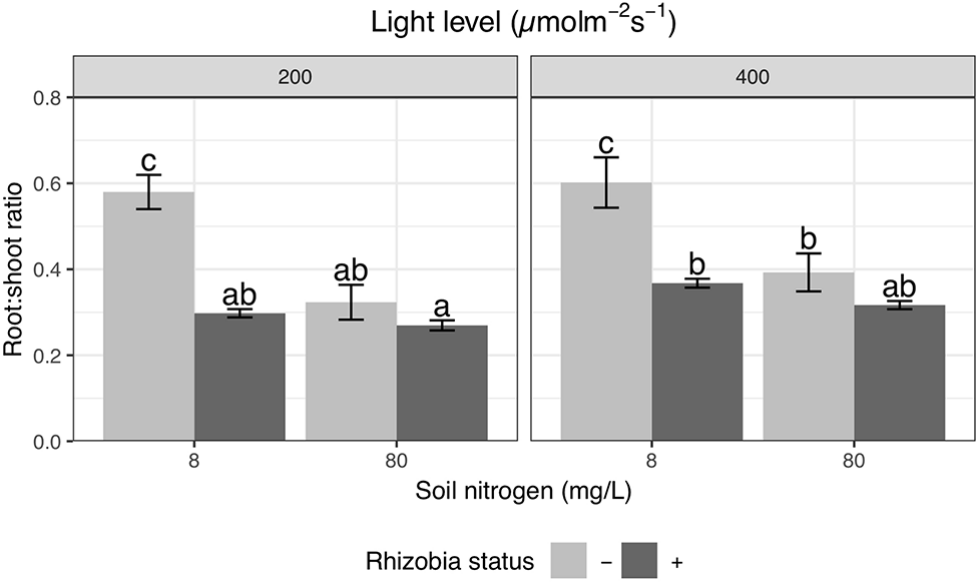
Effects of light, nitrogen, and rhizobia on root:shoot ratio. Error bars represent +/− one standard error (8–11 replicates). Note that root:shoot ratio was ln-transformed to improve normality in the ANOVA but is represented here without transformation for ease of interpretation. Bars with the same letter across the entire figure do not significantly differ after *post hoc* testing with the Tukey test (*P* > 0.05).

Root and shoot biomass were strongly positively correlated with one another (**Figure S1**); yet, we found variation in relative allocation as measured by the root:shoot ratio. High light increases root:shoot ratio by 16% overall (P < 0.01; **Table 4**), though we did not detect a significant effect of light when all pairwise comparisons were tested during Tukey testing (**Figure 3**). We detected a significant interaction between the effects of nitrogen and rhizobia on root:shoot ratio (*P* < 0.0001; **Table 4**). Rhizobia decreased root:shoot ratio by 44% at low soil nitrogen (*P* < 0.001) but had no effect at high soil nitrogen (**Figure 3**). Similarly, increasing soil nitrogen decreased root:shoot ratio by 39% in the absence of rhizobia (*P* < 0.001) but had no significant effect in the presence of rhizobia (**Figure 3**). We note that allocation to nodules within total biomass was remarkably constant and varied sharply between nitrogen levels but not light levels (**Figure S2**).

**Table 4:** ANOVA summarizing the effects of light, nitrogen, rhizobia, and their interaction (*) on root:shoot ratio. Bold indicates statistically significant effects (*P* < 0.05).

	Root:shoot ratio	
	*F_(1, 67)_*	*P*
Light (L)	**8.85**	**0.004**
Nitrogen (N)	**42.2**	**1.2e−08**
Rhizobia (R)	**58.4**	**1.1e−10**
L*N	0.399	0.53
L*R	0.872	0.35
N*R	**16.5**	**1.3e−04**
L*N*R	1.24	0.27

### Light, Nitrogen, and Rhizobia Modulate Plant Performance

**Figure 4:**
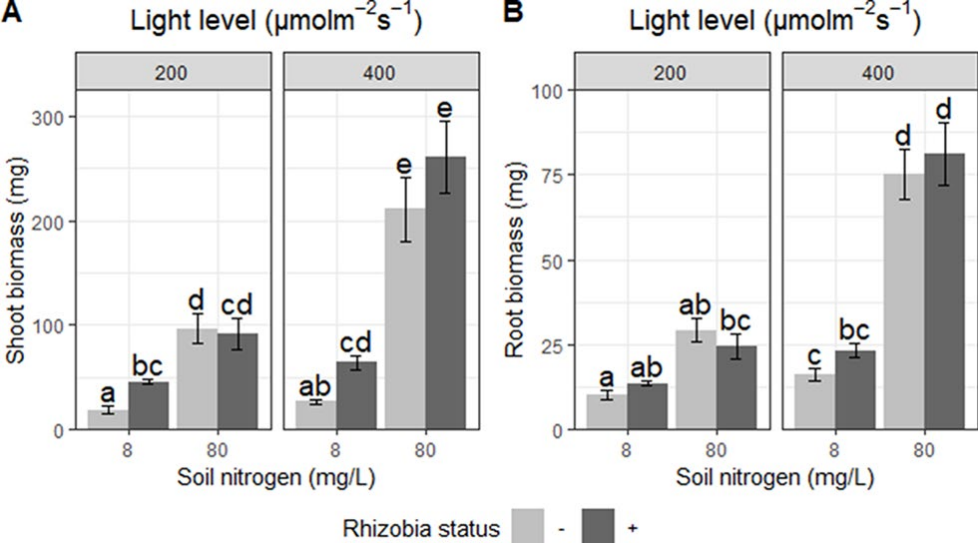
Effects of light, nitrogen, and rhizobia on shoot and root biomass. **(A)** Shoot biomass and **(B)** root biomass of *Medicago truncatula* plants in response to changing soil nitrogen, light availability, and the presence or absence of rhizobia. Error bars represent +/− one standard error (8–11 replicates). Note that root and shoot biomass were ln-transformed to improve normality in the ANOVA but are represented here without transformation for ease of interpretation. Bars with the same letter within an individual panel do not significantly differ after *post hoc* testing with the Tukey test (*P* > 0.05).

We detected significant interactions between the effects of light and nitrogen on shoot biomass (*P* < 0.01; **Table 5**). Light increased shoot biomass by 35% at low soil nitrogen and by 149% at high soil nitrogen (**Figure 4A**). Similarly, increasing soil nitrogen increased shoot biomass by 183% at low light and by 423% at high light (**Figure 4A**). We also detected significant interactions between the effects of soil nitrogen and rhizobia (*P* < 0.0001; **Table 5**). Rhizobia increased shoot biomass by 135% at low soil nitrogen but had no significant effect at high soil nitrogen (**Figure 4A**). Increasing soil nitrogen increased shoot biomass by 581% in the absence of rhizobia but only 207% in the presence of rhizobia (**Figure 4A**).

**Table 5:** ANOVAs summarizing the effects of light, nitrogen, rhizobia, and their interaction (*) on shoot and root biomass. Bold indicates statistically significant effects (*P* < 0.05).

	Shoot biomass		Root biomass	
	*F_(1, 67)_*	*P*	*F_(1, 67)_*	*P*
Light (L)	**41.6**	**1.4e−08**	**71.5**	**3.6e−12**
Nitrogen (N)	**194**	**<2.2e−16**	**132**	**<2.2e−16**
Rhizobia (R)	**27.9**	**1.5e−06**	2.68	0.11
L*N	**8.19**	**0.006**	**11.5**	**0.001**
L*R	0.204	0.65	0.945	0.33
N*R	**19.7**	**3.50e−05**	**6.80**	**0.011**
L*N*R	1.69	0.20	0.646	0.42

Root biomass exhibited similar trends. We detected a significant interaction between the effects of nitrogen and light (*P* < 0.01; **Table 5**). Increasing light increased root biomass by 62% at low soil nitrogen and by 192% at high soil nitrogen (**Figure 4B**). Increasing soil nitrogen increased root biomass by 121% at low light and by 299% at high light (**Figure 4B**). We also found significant interactions between the effects of rhizobia and nitrogen on root biomass (*P* < 0.05; **Table 5**). Rhizobia did not have a significant effect on root biomass, but it changed the magnitude of the effect of soil nitrogen: increasing soil nitrogen increased root biomass by 307% in the absence of rhizobia, but only by 175% in the presence of rhizobia (**Figure 4B**).

## Discussion

In order to understand resource exchange mutualisms, it is critical to know how the resource environment shapes the costs, benefits, and trade decisions of each partner. As predicted, shifting the resource environment altered the plant’s allocation of resources between their rhizobial trading partners and the acquisition of external nutrients. In the biological market framework, increasing soil nitrogen is expected to reduce allocation to trade with rhizobia because the plant is able to obtain a larger proportion of its nitrogen needs through direct nitrogen uptake, which is less energetically costly than nitrogen fixation (Voisin et al., 2002). This is exactly what we observe in response to increased soil nitrogen: plants sharply decreased both their total investment in nodulation (**Figure 1**) and their relative investment to nodules *versus* roots (**Figure 2**). This means that increasing soil nitrogen decreases both nodule initiation and the amount of biomass being allocated to existing nodules. In contrast, *L. strigosus* has been shown to regulate nodulation in response to nitrogen mainly through changes in nodule size (Regus et al., 2015), and soybean has been shown to regulate nodulation in response to nitrogen mainly through changes in nodule number (Lau et al., 2012).

In contrast to trends in the abundance of soil nitrogen, increasing light availability increases the plant’s potential for carbon fixation and thus presumably its carbon supply. This should result in increasing investment into nitrogen acquisition, though the breakdown between direct nitrogen uptake and trade would be based on the relative cost of each, which is determined by the soil nitrogen level (Schwartz and Hoeksema, 1998). We found that increasing light availability increased both total nodule number and total nodule biomass, and this effect was much more pronounced at low soil nitrogen—the only condition in which rhizobia significantly increased shoot biomass (**Figure 1**, **Figure 4**). In addition, light increased the nodule:root biomass ratio only at low soil nitrogen (**Figure 2**). This suggests that plants are investing relatively more biomass into existing nodules with increasing light, but only under nitrogen conditions in which the rhizobia are beneficial to the plant. In contrast, light decreased specific nodulation regardless of nitrogen level (**Figure 2**), which indicates that the increase in total nodule number with increasing light (**Figure 1**) is being driven by an increase in root biomass that overcomes a reduced rate of nodule initiation per unit of root biomass. In total, these complex effects of light suggest that, since the magnitude and direction of the effect of light are different for nodule number and nodule biomass, light may be acting through multiple pathways to regulate nodulation. Furthermore, these results highlight the importance of examining nodulation relative to root biomass when other experimental treatments are expected to alter plant size, while unscaled nodule number and biomass are important predictors of rhizobial fitness (Ratcliff et al., 2012); assessing only unscaled nodulation may lead to misleading conclusions about plant allocation. The wide variation in the effects of light on nodulation reported in the literature may be due to variation in genotype x genotype responses to light, similar to the varying effects of nitrogen detected by Heath et al., 2010, but it may also be explained by differences in the type of nodulation measures reported (i.e., nodule:root biomass in Houx et al., 2009 but unscaled nodule biomass in Santos et al., 1997). Another potentially important factor is the light intensities used. Our experiment, with 200 and 400 µmol•m^−2^•s^−1^, is much lower than full sunlight, which could explain why some of our light effects were not more pronounced.

The benefit that plants get from trade with rhizobia is the product of their investment in rhizobia and the return on investment, or efficiency of the nodules. We measured efficiency as the increase in shoot biomass relative to the control, divided by nodule biomass. We did not detect a significant effect of nodule biomass on shoot biomass gain, but there was generally a positive relationship between the two (**Figure 2**). **Figure 2** also suggests that nodules have a much higher efficiency at high nitrogen than at low, but this is likely an artifact of the visualization. Regardless, there is no support for an effect of light on nodule efficiency as was reported in soybean (Santos et al., 1997; Araujo et al., 2018). There are several possible explanations for this discrepancy, including differences in the magnitude of the change in light, the way efficiency was measured, and the fact that soybeans form determinate nodules while *M. truncatula* forms indeterminate nodules (Denison, 2000). We were only able to measure efficiency as shoot biomass gain per unit of nodule biomass, but Santos et al. (1997) and Araujo et al. (2018) measured it as a milligram of nitrogen fixed per milligram of nodule. Neither measure is perfect because nodule biomass does not account for all of the carbon allocated to rhizobia (Rainbird et al., 1984), and the amount of nitrogen fixed may not directly translate into plant fitness benefit if the biomass yield per nitrogen or relative nitrogen allocation changes. This suggests that *M. truncatula* has the regulation of rhizobial nitrogen fixation very tightly controlled and is always operating it near maximum efficiency, likely due to the energy intensity of nitrogen fixation (Silsbury, 1977; Andrews et al., 2009).

The final aspect of plant biomass allocation is the balance of biomass between the roots (for nitrogen acquisition) and shoots (for carbon acquisition). When we assessed these allocation patterns by measuring the root:shoot ratio, we found strong interactions between the effects of nitrogen and rhizobia. Nitrogen only affected root:shoot ratio in uninoculated plants, and rhizobia only affected root:shoot ratio at low nitrogen (**Figure 3**). Low nitrogen inoculated plants, high nitrogen uninoculated plants, and high nitrogen inoculated plants all had statistically indistinguishable root:shoot ratios (**Figure 3**), even though the low nitrogen plants were significantly smaller than the high nitrogen plants (**Figure 4**). Thus, the rhizobial effect on root:shoot ratio is not fully explained by changes in plant nitrogen status. This suggests that plants are changing their allocation strategy to acquire more carbon to trade for nitrogen instead of directly acquiring nitrogen with root biomass. Goh et al. (2016) used a strain that forms nodules but does not fix nitrogen to show that rhizobial effects on root:shoot ratio in *M. truncatula* appear to depend only on nodule initiation, not on nitrogen fixation by the rhizobia. Thus, this trend may show that rhizobia are able to manipulate plants into allocating more resources to trade even when it is not beneficial or that root:shoot allocation decisions become fixed and do not change as a function of the actual nitrogen-fixation levels of nodules.

Finally, our data show that increased light strengthens the effect of increasing soil nitrogen on plant growth but does not make rhizobia more beneficial, likely due to the higher cost of obtaining nitrogen through symbiosis. We detected significant interactions between nitrogen and rhizobia effects and between nitrogen and light effects on shoot biomass, but not between light and rhizobia (**Table 5**). Rhizobia are not beneficial to the plant at high soil nitrogen levels because of the high cost of nitrogen fixation (Silsbury, 1977; Voisin et al., 2002; Andrews et al., 2009) and the ability of the plant to obtain sufficient “cheap” nitrogen directly from the soil. The interaction of nitrogen and light can be explained by the plant being strongly nitrogen limited at low soil nitrogen levels, so that increasing light does not allow more growth because the limiting resource is nitrogen. However, at high soil nitrogen and low light, carbon is limiting, so increasing light availability allows for a much larger nitrogen effect. At first, it seems counterintuitive that investment in rhizobia is increased by increasing light, and that efficiency does not change depending on conditions, but the benefit from rhizobia does not change depending on light. This is likely due to the high cost of nitrogen fixation: since each unit of nitrogen fixed by rhizobia costs a relatively high amount of carbon (Silsbury, 1977; Voisin et al., 2002; Andrews et al., 2009); this change in allocation does not have statistically significant effects on shoot biomass. In addition, the carbon costs of rhizobia may be at least partially counteracted by the stimulation of photosynthesis by nodules, either by improved leaf nitrogen status (Kaschuk et al., 2009) or through the extreme sink strength of nodules (Brown and Bethlenfalvay, 1987; Kaschuk et al., 2009; Kaschuk et al., 2010; Kaschuk et al., 2012), meaning that these mechanisms may counteract the carbon dependency of benefit from nodules. At higher light levels, if plants have excess carbon that they are not able to convert into biomass due to nitrogen limitation, this may be fed to nodule-occupying rhizobia or exuded into the rhizosphere, analogous to aphids that can secrete a large fraction of the sucrose they consume when they are nitrogen-limited (Mittler and Meikle, 1991).

It is important to note that, due to limitations of the facilities and equipment available, the light levels used in this experiment were a relatively small fraction of the maximum light intensity a plant might experience in full sun [200–400 µmol•m^−2^•s^−1^ compared to 2,000 µmol•m^−2^•s^−1^; (Korczynski et al., 1991)]. However, these levels are within the range that legumes may face when being shaded by other plants (Burkey and Wells, 1991), and span a large portion of the suggested range of 200–600 µmol•m^−2^•s^−1^for growing *Medicago* in the growth chamber (https://www.noble.org/medicago-handbook/). Light level had a significant impact on plant biomass accumulation (**Figure 4**, **Table 5**), suggesting that light is limiting under these conditions, even though it did not have as large an effect as our nitrogen manipulation. One important caveat of this work is the fact that manipulating light levels has broader effects beyond simply altering total carbon available to the plant. Changes in light availability, such as what plants in vegetation canopies experience, induce changes in traits such as shoot architecture (Cescatti and Niinemets, 2004; Poorter et al., 2009) and chlorophyll content (Evans, 1993). The reduction in photosynthesis and thus growth due to low light reduces demand for soil nutrients (Cui and Caldwell, 1997). However, manipulating light levels offers a highly feasible, ecologically relevant method of altering nutrient availability to examine its effects on mutualisms. Understanding the effect of shading on mutualisms is agriculturally important for the use of legumes in agroforestry (Houx et al., 2009) and for determining optimum planting density in commercial soybean crops (Pons and Pearcy, 1994).

## Conclusions

In this study, we demonstrated interactive effects of light and nitrogen on plant investment into and benefit from trade with rhizobia. It will be particularly important to conduct these tests in other plant-microbe systems to determine whether optimal allocation patterns differ between various plant-microbe symbioses. There has been extensive theoretical work regarding the context dependence of the benefits of resource mutualisms, but empirical work in this field has been limited. Thus, the area offers a unique opportunity to combine theoretical work and empirical testing to further our understanding of mutualism function and stability across evolutionary time (Clark et al., 2017). This avenue of research will have important implications for predicting how anthropogenic impacts on non-substitutable nutrient availabilities will affect plant-microbe symbioses both in agricultural and natural environments.

## Data Availability Statement

All datasets generated for this study are included in the article/**Supplementary Files**.

## Author Contributions

CF and MF designed the research. CF performed experiments and analyzed data with guidance from MF. CF and MF wrote the manuscript.

## Conflict of Interest

The authors declare that the research was conducted in the absence of any commercial or financial relationships that could be construed as a potential conflict of interest.

## Supplementary Material

Table S1 Raw data used in this manuscript.

Table S2 Explanation of column headers in Table S1.

Figure S1 Plant root biomass (g) plotted against shoot biomass (g). Colors indicate treatment conditions. Each point represents an individual plant.

Figure S2 Ternary plots depicting the percentage of total plant biomass allocate to shoots, roots, and nodules. Each point represents an individual plant.
