# Supplementary figures and images for: Legumes Modulate Allocation to Rhizobial Nitrogen Fixation in Response to Factorial Light and Nitrogen Manipulation
Source: Front Plant Sci. 2019 Nov 5;10:1316. doi: 10.3389/fpls.2019.01316 (PMC6848274; doi:10.3389/fpls.2019.01316)

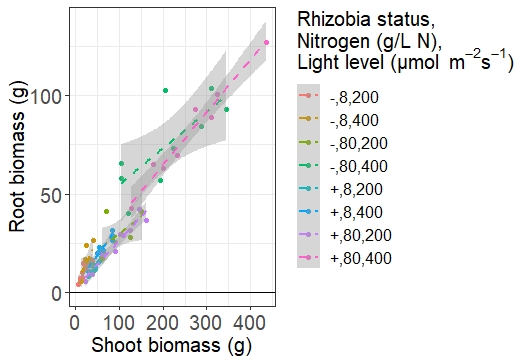

Supplement: Figure S1 — Plant root biomass (g) plotted against shoot biomass (g). Colors indicate treatment conditions. Each point represents an individual plant. [file Image_1.jpeg]

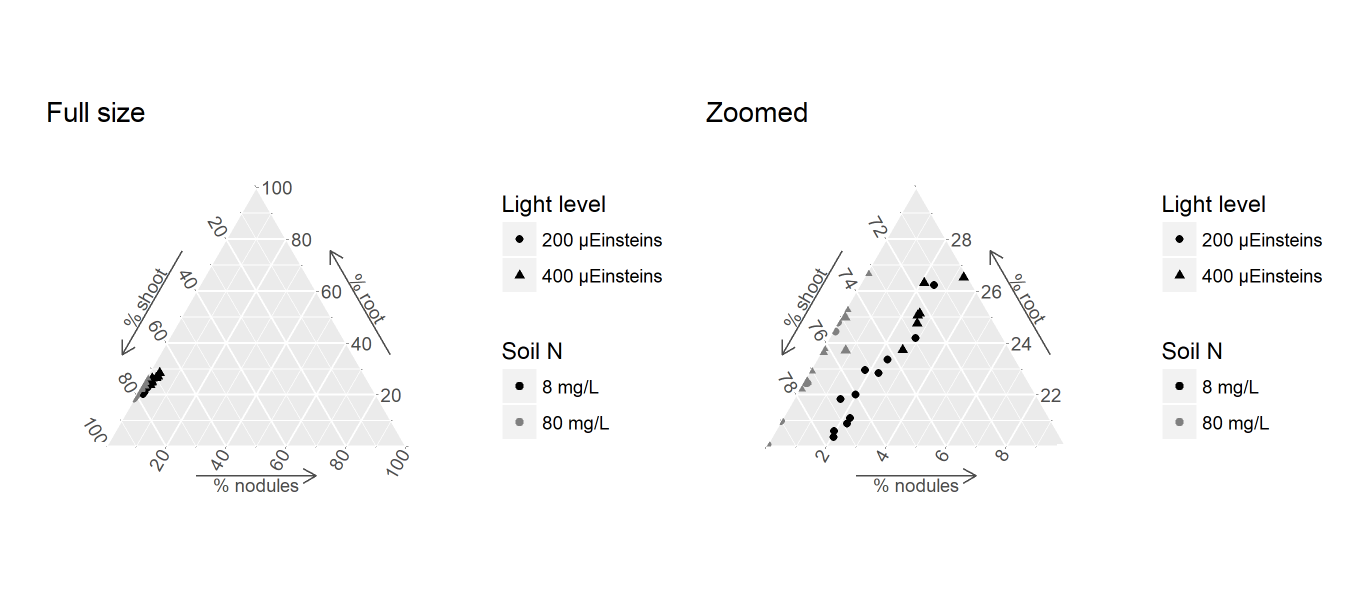

Supplement: Figure S2 — Ternary plots depicting the percentage of total plant biomass allocate to shoots, roots, and nodules. Each point represents an individual plant. [file Image_2.tif]
